# Outstanding questions in the study of archaic hominin admixture

**DOI:** 10.1371/journal.pgen.1007349

**Published:** 2018-05-31

**Authors:** Aaron B. Wolf, Joshua M. Akey

**Affiliations:** 1 Department of Genome Sciences, University of Washington, Seattle, Washington, United States of America; 2 Department of Ecology and Evolutionary Biology, Princeton University, Princeton, New Jersey, United States of America; 3 Lewis-Sigler Institute, Princeton University, Princeton, New Jersey, United States of America; Stanford University School of Medicine, UNITED STATES

## Abstract

The complete sequencing of archaic and modern human genomes has revolutionized the study of human history and evolution. The application of paleogenomics has answered questions that were beyond the scope of archaeology alone—definitively proving admixture between archaic and modern humans. Despite the remarkable progress made in the study of archaic–modern human admixture, many outstanding questions remain. Here, we review some of these questions, which include how frequent archaic–modern human admixture was in history, to what degree drift and selection are responsible for the loss and retention of introgressed sequences in modern human genomes, and how surviving archaic sequences affect human phenotypes.

## Introduction

Today, humans are the only hominin species walking the planet. This exclusivity is a recent feature of our species’ history. Specifically, though anatomically modern humans first appear in the archaeological record 200–300 kya [1–4], other hominins persisted until as recently as 30–40 kya [5,6]. In some cases, modern humans overlapped temporally and spatially with archaic humans (for example, Neanderthals and Denisovans and perhaps others [7,8]). Neanderthals left a rich archaeological and paleontological record and resided in the Middle East, Europe, and parts of Asia [9–11]. Denisovans, whom we only know about from ancient DNA taken from a single finger bone and three teeth [12–14], are believed to have resided in parts of East and Southeast Asia.

There has been long-standing interest in whether anatomically modern humans and archaic human ancestors hybridized. Historically, attempts at answering this question focused on archaeological remains and compared dental, cranial, and postcranial features from modern human and archaic human sites for evidence of hybrid morphologies [15]. By the early 2000s, technological innovations enabled the extraction and sequencing of mitochondrial DNA from archaic human remains [16–19] and eventually facilitated the capture and sequencing of the full nuclear genome [20–22].

The complete sequencing of archaic and modern human nuclear genomes led to the discovery that modern non-African human populations shared more genetic ancestry with archaic humans than did African populations [22]. Initial inferences demonstrated a strong likelihood of hybridization between archaic humans and the ancestors of all modern non-African populations, and these results proved robust to alternative explanations, such as archaic population structure. The continued development of ancient DNA technology facilitated extraction and sequencing of high-quality Neanderthal [23] and Denisovan [13] reference genomes. These foundational resources, coupled with advances in statistical and computational tools for analyzing ancient genomes, enabled the identification of sequences inherited from archaic ancestors (i.e., introgressed sequences) in the genomes of modern human individuals.

Considerable progress has been made in the study of archaic–hominin admixture, which has been reviewed elsewhere [24–28]. However, many outstanding questions remain, the resolution of which are critical to more completely understand the history and consequences of admixture between archaic and modern humans. In this review, we discuss several of these questions, including refining models of admixture history, determining the mechanisms responsible for the loss and retention of archaic sequence, and describing the functional implications of surviving Neanderthal sequence in the modern human genome.

## How high was the initial level of archaic–modern human admixture?

All modern non-African genomes are estimated to carry approximately 2%–7% archaic human sequence: approximately 2% ancestry from Neanderthals and an additional 2%–5% ancestry from Denisovans in Melanesian populations [29–31]. However, present-day levels of archaic ancestry need not reflect initial admixture levels, which is of special interest in understanding human history. Specifically, an accurate estimate of initial admixture levels would provide significant insights into models of hybridization and admixture dynamics.

Following the discovery of approximately 2% Neanderthal ancestry in modern non-Africans [22], it was estimated the initial level of admixture between Neanderthals and modern humans was also 2% [32–34]. However, further analyses revealed large depletions of Neanderthal ancestry across the human genome, suggesting widespread purging of deleterious Neanderthal sequence. For example, in the 20% of the genome with the lowest density of functionally important elements, Neanderthal ancestry is 1.54× the genome-wide average [34]. Assuming this subset of the genome to be unaffected by selection, the implication is that the initial proportion of Neanderthal ancestry after admixture was >3%.

Several recent analyses have estimated the initial Neanderthal admixture proportion was dramatically higher than 3% [35,36]. These studies propose the prolonged small effective population size of Neanderthals led to a high frequency of weakly deleterious alleles in the Neanderthal population [35,36]. When these Neanderthal alleles entered the human population, with a comparatively larger effective population size, they were more readily removed by selection. Using simulations and models reflecting this expectation, these studies estimate the initial admixture proportion to have been 2×–5× the level present in modern human genomes.

Analyses of additional ancient DNA samples support the hypothesis that initial admixture levels were much higher than those found today. Genome-wide data from Eurasian samples ranging in age from 45–7 kya suggest an initial Neanderthal admixture proportion close to 6%, which decreased gradually over time to a contemporary level of 2% [37]. Because all the individuals analyzed descended from a single founding population, the authors argue the steady decline in Neanderthal ancestry is driven by natural selection against introgressed sequence and not dilution from a nonadmixed population. Consistent with this hypothesis is the discovery of an ancient East Asian individual, dated to 40 kya, who was an ancestor of modern Asians and who carried 4%–5% Neanderthal ancestry [38]. Additionally, sequence data from a 42 kya anatomically modern human from Pęstera cu Oase, Romania, reveal this individual shared 6%–9% of his genome with Neanderthals, more than 3× any contemporary modern humans [39]. However, it is important to note that the Pęstera cu Oase individual had a very recent Neanderthal ancestor (within 4–6 generations) and likely did not contribute any ancestry to modern populations.

If the initial proportion of Neanderthal ancestry was indeed higher, it raises additional questions about the rate at which Neanderthal sequence was lost. Though analysis of ancient samples projects a gradual and linear reduction in Neanderthal ancestry from 45 kya to the present [37], simulations involving high frequency and weakly deleterious alleles indicate a process featuring an initial rapid loss of Neanderthal sequence followed by a more gradual loss [35]. Answering questions regarding the initial admixture level, the duration of admixture, and the rate of archaic-sequence loss will depend on the continued collection of ancient human and Neanderthal samples closer to the time of admixture. To date, no estimates have been provided for initial levels of Denisovan admixture, in part because of the paucity of data and the uncertainty regarding where and when admixture could have occurred.

## How many distinct pulses of admixture occurred with Neanderthals?

**Fig 1:**
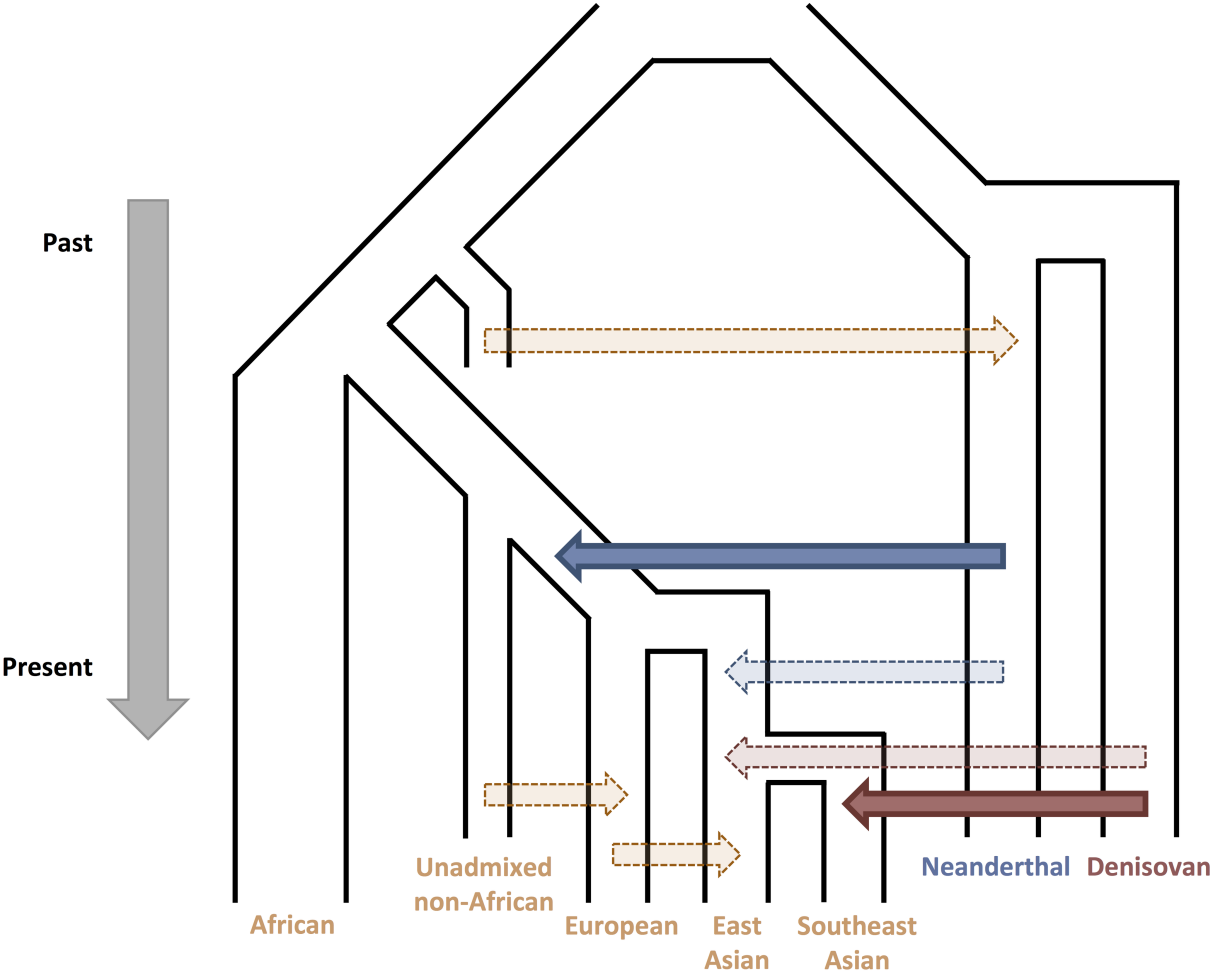
A simplified model of admixture history between archaic and anatomically modern human populations. There is consensus that at least two independent gene-flow events occurred (solid arrows)—admixture from Neanderthals into an ancestral Eurasian population (solid blue) and from Denisovans into an ancestral Southeast Asia population (solid red). It is likely that additional instances of admixture occurred, explaining the variation in the percentage of archaic sequence across different global populations. These additional instances include a pulse of admixture from Neanderthals (dashed blue) and from Denisovans (dashed red) into an ancestral East Asian population. Alternatively, or in addition, global variation in archaic ancestry could be the result of admixture within human populations (dashed orange) diluting archaic sequence. Admixture from human populations may also have introduced sequence into archaic populations.

Closely related to the question of how high initial levels of admixture were, the number of independent admixture events (sometimes referred to as “pulses” in the literature) is also uncertain. Initially, this question seemed to have a simple answer—admixture with Neanderthals occurred once in human history. Early studies found that all non-Africans carried approximately equal levels of Neanderthal ancestry [22]. Therefore, the most parsimonious model involved a single pulse of admixture between Neanderthals and an Out-Of-Africa wave of human migrants, before the ancestral Eurasian population split into European and Asian lineages (Fig 1).

**Fig 2:**
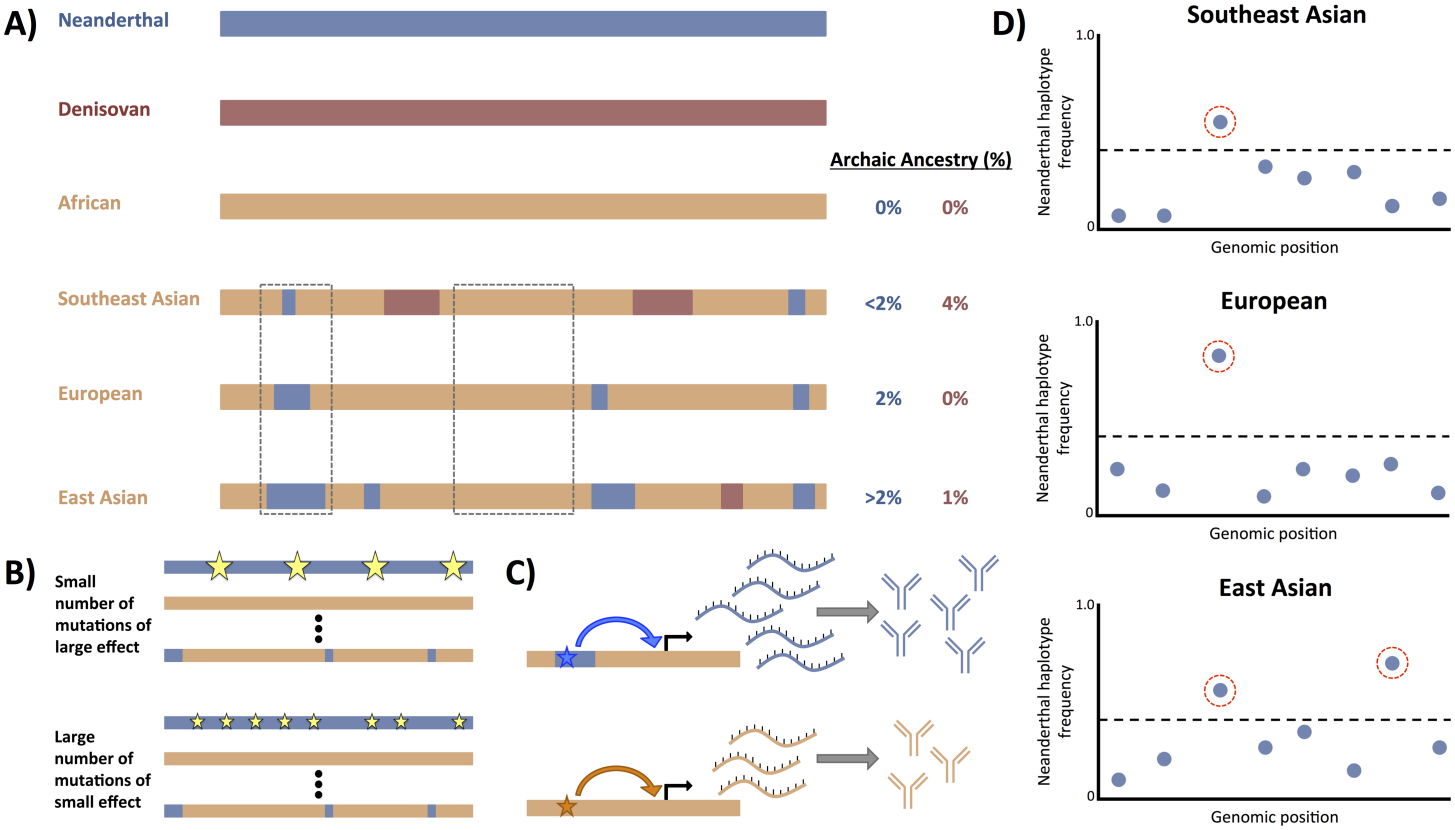
Patterns and characteristics of archaic sequence across the genome. (A) A representation of individual genomes from archaic and modern human populations. The modern human genomes (orange) are ordered by increasing levels of Neanderthal (blue) admixture percentage (approximate). Only Asian populations carry Denisovan (red) sequence. Some introgressed archaic segments are shared across populations, and some large regions of the genome are deserted of introgressed archaic sequence in all populations examined. (B) Large deserts may be a product of selection against deleterious archaic variants (gold stars) at those loci. Whether selection acted against a few strongly deleterious variants (top) or many weakly deleterious variants (bottom) remains uncertain. (C) Many segments of introgressed archaic sequence are found to carry variants (stars) that affect gene regulation and expression. Altering gene expression may affect downstream protein levels (e.g., immunological proteins) and could have provided a mechanism of rapid adaptation for admixed modern humans. (D) Putatively adaptive introgressed segments can be identified by examining the frequency of introgressed segments (blue dots) within a population and filtering for those that exceed a percentile cutoff (dashed black line).

However, as more globally diverse populations were analyzed with refined methods, they found that levels of Neanderthal ancestry varied among populations. Analyses of introgressed Neanderthal sequence using the high-quality Altai reference genome [23] noted more regions of Neanderthal origin in Asian and American populations than European ones [32], as well as higher levels of Neanderthal ancestry in East Asian populations compared to European populations and lower levels of Neanderthal ancestry in Melanesians compared to either East Asians or Europeans [30,31] (Fig 2A). The differences in these Neanderthal ancestry proportions are on the order of 0.1%–0.5%. How we interpret the global variation in archaic human ancestry has a profound impact on our understanding of human history, informing our estimate for the frequency of archaic–modern human admixture—either as isolated in time and space or recurrent and pervasive.

Considering the variation in levels of Neanderthal ancestry among populations, a single pulse of admixture may still be the most parsimonious explanation. For example, admixture between ancient Europeans and populations lacking Neanderthal ancestry could have diluted the amount of Neanderthal ancestry remaining in modern European populations [13,40] (Fig 1). It has also been proposed that less-efficient purifying selection in East Asians due to a smaller effective population size led to the retention of more Neanderthal sequence [34].

Alternatively, several analyses using statistical and simulation approaches suggest that models incorporating multiple pulses of admixture better explain the data [30,33,41–43]. These models include an initial wave of admixture into an ancestral non-African population, followed by additional admixture events into an ancestral Eurasian population and ancestral East Asian population [43] or just an ancestral Asian population [33] (Fig 1). Studies simulating admixture over a range of selection and demographic models can only account for the higher proportion of Neanderthal ancestry in East Asians compared to Europeans by including multiple pulses of admixture [42,44]. Even a “two-pulse” model may be too simplistic a representation for the history of human and Neanderthal admixture. Simulations that included additional admixture events, such as an intermediate admixture pulse into the ancestral population of Europeans and East Asians but not Southeast Asians, are also compatible with the empirical data for archaic ancestry in diverse populations [30]. It is important to note that the estimated number of admixture “pulses” is an oversimplification of real population history assumed for modeling, and compatible with a broad range of specific gene-flow models with varying intensities and duration.

## Was there a sex bias in gene flow?

Sex biases—historical differences in sex ratios—are common in modern human populations, and several studies have shown them to vary across time using sequence data collected from the X chromosomes of geographically diverse populations [45–47]. Sex-biased hybridization has been invoked, along with other mechanisms, as an explanation for the reduced level of Neanderthal ancestry along the X chromosome—approximately one-fifth that of the autosomes [33,34]. Models of hybridization that specifically examined the X chromosome found the range of selection values against Neanderthal sequence varied for simulated Asian and European populations—possibly a result of differing effective population sizes—and that selection against Neanderthal sequence on the X chromosome was greater than on the autosomes [36]. The difference in the strength of selection against Neanderthal sequence on the X chromosome versus the autosomes may also be, in part, influenced by the hemizygosity of X chromosome genes in males.

Alternatively, reduced Neanderthal ancestry on the X chromosome is also consistent with models that use a lower initial admixture proportion for the X chromosome, reflecting a bias towards more frequent Neanderthal male and human female pairings—potentially as great as 3× more frequent than the complementary pairing [36]. A bias towards Neanderthal male and human female pairings could also help explain why investigations of Neanderthal and human mitochondrial DNA, which is inherited maternally, show no indication of Neanderthal–human admixture [19,48]. Questions surrounding sex-biased hybridization—if it occurred, its direction and magnitude, and whether it varied among non-African populations with different admixture histories—could yield new insights into the cultural dynamics of archaic admixture. Furthermore, the above studies have focused on instances of Neanderthal gene flow into human populations, while the reciprocal situation—human gene flow into Neanderthal populations—may show a different sex bias because of biological mechanisms, cultural practices, or historical events.

## Who were the Denisovans?

While there exists a wealth of archaeological data regarding the geographic distribution of Neanderthals, informing models of where and when admixture could have occurred, there is a paucity of corresponding data for the other known archaic human species—Denisovans. This leaves us with a limited ability to model where admixture occurred, into which populations, and how frequently. The fact that the only Denisovan remains were found in Siberia, while the population carrying the greatest Denisovan ancestry resides in Melanesia [12,49,50], suggests the process by which Denisovan DNA entered Asian populations was complex. Does the substantial distance between the Denisova Cave and Southeast Asia indicate that Denisovans inhabited such a large geographic range? If so, why do geographically intermediate populations in East Asia carry a substantially smaller proportion of Denisovan ancestry?

Models aiming to explain the distribution of Denisovan ancestry have invoked either multiple pulses of admixture into separate East and Southeast Asian lineages, each undergoing unique demographic events [50], or dilution of Denisovan ancestry in East Asian lineages through admixture with populations lacking any Denisovan ancestry [51,52] (Fig 1).

Discordance between the Denisovan and modern human divergence times estimated from mitochondrial DNA and nuclear DNA—1 million years ago versus 500,000 years ago, respectively—has been interpreted as evidence that Denisovans also interbred with another archaic hominin distinct from Neanderthals or humans [12,53]. Possible candidates for such interbreeding include *Homo erectus* and *H*. *heidelbergensis*, archaic human species that archaeological data suggest inhabited East and Southeast Asia as recently as 100 kya [54,55]. Might these archaic species have also interbred with anatomically modern humans? An accurate model of Denisovan and human admixture and a better understanding of Denisovans in general heavily depend on finding more Denisovan and other archaic human species remains.

## Did archaic hominin admixture happen in Africa?

While the genetic evidence, collected from archaic and modern human DNA samples, persuasively demonstrates archaic hominin admixture in non-African populations, similar studies of archaic admixture in African populations have been limited. This is despite the fact that numerous archaic hominin lineages are known to have existed in Africa [56] and may have overlapped in time and space with modern humans [57]. Studies of archaic admixture in Africans have been hindered by the historical underrepresentation of African populations in large genomic datasets and the absence of reference genomes for archaic African hominins—the combined effects of the greater age of archaic samples and challenging climate impeding the recovery of ancient DNA.

Several studies, however, have made a concerted effort to investigate the likelihood of archaic admixture in African populations, leveraging linkage-disequilibrium–based [58,59] and demographic-model–based [60] methods for detecting signals of archaic admixture without an archaic reference genome. Evidence from these early studies does indicate admixture occurred between an unidentified archaic hominin ancestor and several African populations and contributed functionally relevant genetic variation at specific loci, such as the salivary *MUC7* locus [61]. In the absence of any recovered ancient DNA samples, “excavating” archaic sequences from modern African genomes may be the best strategy to identify archaic hominin lineages. Although studies of archaic admixture in Africa are limited and have been necessarily cautious in their conclusions, we anticipate significant new discoveries as more genomic data from diverse African populations become available.

## What caused deserts of archaic sequence to form?

Compiling the surviving introgressed archaic human haplotypes in hundreds of individuals from geographically diverse populations led to a “map” of introgressed sequence across the human genome. While introgressed sequence tends to be widespread across the genome, covering all 22 autosomes and the 2 sex chromosomes, it was a striking discovery to find that there also exist large depletions—“deserts”—of archaic ancestry (Fig 2A).

On the autosomes, the largest deserts span multiple megabases, with a handful extending up to 10 Mb in length [31,33]. The mechanisms responsible for the heterogeneous distribution of Neanderthal sequence across the autosomes are not yet fully understood, and several may act in combination. Understanding the processes responsible for this heterogeneous distribution could be informative about what distinguished modern and archaic humans.

One proposed explanation for autosomal deserts is that they resulted from intense bottlenecks in the human population [34]. Theoretically, a bottleneck soon after admixture with Neanderthals could cause the rapid loss of large introgressed haplotypes, before they could be broken apart by generations of recombination. Simulations exploring some of these extreme demographic scenarios have found genetic drift able to explain some, but not all, of the observed data [30].

Alternatively, selection against Neanderthal haplotypes at desert loci might also generate large depletions of archaic sequence. Selection against specific deleterious Neanderthal alleles in the admixed population could remove large swaths of linked archaic sequence. Deserts of introgressed sequence do exhibit higher levels of background selection and human–Neanderthal sequence divergence [30,33]. Furthermore, deserts of Neanderthal sequence overlap with deserts of Denisovan sequence significantly more often than expected by chance [30]. These data indicate the repeated loss of archaic DNA at specific loci across multiple independent admixture events.

If selection played a part in the removal of large Neanderthal haplotypes and the formation of deserts, an obvious question is whether selection acted strongly on a very few sites or weakly across multiple sites (Fig 2B). Studies modeling the effective population sizes of Neanderthals and humans before and during admixture suggest that the small size of the Neanderthal population would have allowed weakly deleterious alleles to drift as if neutral and accumulate at a high frequency [35]. When these alleles entered the human population through admixture, the effective size of the human population need only have been marginally larger than the Neanderthal population to increase the strength of selection against these alleles and effect their removal. At the same time, deserts of introgression tend to exhibit higher levels of background selection and are also significantly enriched for genes expressed in the brain, such as *FOXP2*, which is essential to speech and language development [30,33,62]. These patterns suggest strong selection at a single locus could drive the loss of Neanderthal sequence across a wide region. Furthermore, environmental differences between modern and archaic humans may have meant that, rather than just the force of selection changing, the selection pressures themselves might have changed for archaic alleles when they entered the human population. What was potentially advantageous or neutral in a Neanderthal population may have been deleterious in a human one.

Finally, it is important to note explanations beyond drift and selection in forming deserts. For instance, large inversions on either the human or Neanderthal and Denisovan lineages could theoretically prevent introgression in these regions by suppressing recombination. Considering the overlap of Neanderthal and Denisovan deserts [30], large inversions seem unlikely to explain all of the archaic depletions found to date but remain a formal possibility. Unfortunately, identifying potential lineage-specific inversions is incredibly difficult given the deterioration of ancient DNA samples and short sequencing read lengths.

## What are the functional and phenotypic consequences of hybridization?

A critical question in studying archaic–modern human hybridization is the functional impact of the remaining introgressed archaic sequence in the modern human genome. How has introgressed sequence shaped human evolution? How is it currently affecting modern human phenotypes and health and disease? Is the effect of Neanderthal sequence on human phenotypes proportional to the low amount of Neanderthal ancestry present in the human genome, or are there instances where Neanderthal ancestry has a disproportionately large effect?

Several studies examining certain modern human populations have identified introgressed Neanderthal haplotypes that have risen to higher frequency than expected by drift (Fig 2D). The functional significance of these genes has been hypothesized based on prior biological studies and association with normal and disease phenotypes [63,64]. For example, a Neanderthal version of the gene *BNC2* was identified at high frequency in several non-African populations—a sign of putative adaptive introgression—and is associated with skin pigmentation levels in Europeans [63,65]. Additionally, putatively adaptive introgressed sequences have been identified at several genes that play key roles in immunological function, such as *STAT2* [66], *OAS1* [67], and *TLR1/6/10* [65,68]. There are also examples of certain populations carrying putatively adaptive introgressed sequences from Denisovans, such as in Greenlandic Inuit the genes *TBX15* and *WARS2* [69]—associated with adipose tissue differentiation and distribution—and in Tibetans the high-altitude adaptation gene *EPAS1* [70]. These and other instances of possible adaptive introgression [71] support the hypothesis that archaic hominins, who inhabited Eurasia for 400 ky before humans, would have been a source of advantageous genetic variants pre-adapted to local environmental features, such as colder climates, lower ultraviolet (UV) exposure, and endemic pathogens. Alternatively, archaic hominins may simply have provided a reservoir of additional genetic variation to modern humans, some of which happened to be advantageous following introgression into modern humans but which were not necessarily pre-adapted to the Eurasian environment. It should also be noted that selection against introgressed archaic alleles seems to have been the predominant pattern across the genome as indicated by the low levels of retained archaic ancestry, especially in functionally important regions. This raises questions of whether admixture was beneficial overall for ancient humans migrating out of Africa and to what extent the benefits of some alleles were able to outweigh the costs of others.

While there is increasing power to detect these archaic introgressed segments in modern human populations, our understanding of the evolutionary and fitness consequences remains murky. In the case of introgressed Neanderthal sequence, researchers have leveraged large association studies to infer the effects of archaic sequences [63,64]. Applying a similar approach to introgressed Denisovan sequences has proven more difficult, since those populations with Denisovan ancestry are underrepresented in large association studies and genomic data sets. Furthermore, if our intent is to understand the features under selection at the time of introgression, it is important to remember that the phenotypic effects of archaic sequence we see manifested today appear in very different environments than the ones for which they would have been selected. This will confound attempts to draw connections between the phenotypic effects of introgressed archaic sequences today and the original selected phenotypes.

In addition to leveraging association studies or prior biological findings to infer the effects of introgressed sequence, several recent studies have examined the direct effects of introgressed variants on gene regulation. At sites of putatively adaptive introgressed archaic sequences, researchers have observed Neanderthal alleles affecting expression levels of immunologically relevant genes *OAS1/2/3* and *TLR1/6/10* and observed that these expression differences can be cell-type specific and influenced by environmental stimuli [65]. Others have correlated the genotypes of putatively introgressed Neanderthal alleles with the expression of nearby genes and found introgressed archaic alleles contribute proportionally more to expression variation than nonarchaic alleles [72]. In individuals that are heterozygous for the Neanderthal and human alleles, researchers found frequent instances of allele-specific expression and a significant down-regulation of Neanderthal allele expression in specific brain subregions and the testes relative to other tissues [73]. These findings suggest the phenotypic effects of introgressed archaic sequences are more likely mediated through gene regulation than protein changes (Fig 2C). Recent developments in genomic editing technologies should allow future studies to more thoroughly explore these regulatory effects through in vitro experiments.

## Was there gene flow from modern humans into Neanderthals?

Most studies to date have focused on the Neanderthal and Denisovan contribution to the modern human genome through hybridization. However, investigating the contribution of modern human admixture to these archaic hominin genomes is also of great interest. New research is uncovering instances of potential gene flow from early humans into Neanderthal populations. Analyses of nuclear DNA from multiple Neanderthal samples and modern humans [74] support models in which an early human population—diverged from the population ancestral to contemporary Africans and non-Africans—contributed low levels (0.1–2%) of sequence to a Neanderthal lineage ~100 kya (Fig 1). More complete data from these archaic DNA samples [75] improved these estimates, demonstrating the gene flow event occurred at least 130–145 kya into a lineage ancestral to both Vindija and Altai Neanderthal populations. Analyses of mitochondrial DNA from multiple Neanderthal and human samples suggest that an even earlier gene flow occurred from humans into Neanderthals, potentially as early as ~300 kya [76]. These studies highlight the importance of collecting and analyzing additional archaic samples as well as illustrating the complexity and likely pervasiveness of admixture between different hominin groups.

Characterizing human gene flow into archaic hominins allows researchers to explore novel hypotheses, for example, the potential for sex bias in Neanderthal populations. As noted previously, the depletion of Neanderthal ancestry on the modern human X chromosome could be the product of more frequent Neanderthal male and human female pairings leaving descendants in the human population. If a reciprocal depletion of human ancestry was found on the Neanderthal X chromosome, it could indicate a greater frequency of human male and Neanderthal female pairings in the Neanderthal population. Such findings could be informative about early cultural dynamics, for instance, if admixed offspring were more likely to remain with the mother’s population than the father’s.

## Conclusion

The complete sequencing of archaic and modern human genomes and the discovery that all non-African populations carry ~2% Neanderthal ancestry was a significant breakthrough in anthropology and paleogenomics. Subsequent studies have expanded on this research, cataloguing a richly complex history of human admixture, migration, and evolution. Despite this, many questions still remain about the functional implications of surviving archaic sequences in the modern human genome, the role selection has played in the retention and loss of introgressed sequences, and what the extent of admixture was between archaic and modern humans. Future analyses using improved methods to detect introgressed archaic sequence in more geographically diverse modern populations—whose unique population histories may mean they carry distinct archaic introgressed haplotypes—will be able to answer some of these questions.

Significant progress, however, requires the analysis of additional archaic samples—Neanderthals, Denisovans, and others—and additional ancient human samples dated closer to the time of admixture. Data from more archaic samples [77] improve our understanding of Neanderthal and Denisovan genetic diversity, population structure, and the frequency of admixture events with anatomically modern humans. The analysis of a new, high-coverage Neanderthal genome from Vindija Cave [75] has improved estimates of the Neanderthal effective population size, determined the admixing Neanderthal population to be closer to the Vindija Neanderthal populations than the Altai one, and marginally increased estimates of Neanderthal ancestry in non-African populations outside Oceania to 1.8–2.6%. Analyzing older human samples, closer to the time of admixture, will be informative about the true initial level of admixture as well as the rate at which archaic sequence was lost and thereby provide insight into the mechanisms responsible for the loss and retention of archaic sequence in the modern human genome. As methods and technologies improve our ability to extract and sequence ancient DNA samples, we believe answers to these outstanding questions will soon be revealed.
